# Chloro-benquinone Modified on Graphene Oxide as Metal-free Catalyst: Strong Promotion of Hydroxyl Radical and Generation of Ultra-Small Graphene Oxide

**DOI:** 10.1038/srep42643

**Published:** 2017-03-28

**Authors:** He Zhao, Juehua Wang, Di Zhang, Qin Dai, Qingzhen Han, Penghui Du, Chenming Liu, Yongbing Xie, Yi Zhang, Hongbin Cao, Zhuangjun Fan

**Affiliations:** 1 Beijing Engineering Research Center of Process Pollution Control, Division of Environment Technology and Engineering, Institute of Process Engineering, Chinese Academy of Sciences, Beijing 100190, China; 2 University of Chinese Academy of Sciences, Beijing 100049, China; 3 State Key Laboratory of Multiphase Complex System, Institute of Process Engineering, Chinese Academy of Sciences, Beijing 100190, China; 4 Key Laboratory of Superlight Materials and Surface Technology, Ministry of Education, College of Material Science and Chemical Engineering, Harbin Engineering University, Harbin 150001, Heilongjiang, China

## Abstract

Carbon-based metal-free catalyst has attracted more and more attention. It is a big challenge to improve catalytic activity of metal-free catalyst for decomposition of H_2_O_2_ to produce hydroxyl radical (HO•). Here, we report chloro-benquinone (TCBQ) modified on graphene oxide (GO) as metal-free catalyst for strong promotion of HO•. By the incorporation of GO, the HO• production by H_2_O_2_ and TCBQ is significantly promoted. Based on density functional theory, TCBQ modified GO (GO-TCBQ) is more prone to be nucleophilic attacked by H_2_O_2_ to yield HO• via electron transfer acceleration. Furthermore, the generated HO• can cut GO nanosheets into uniform ultra-small graphene oxide (USGO) through the cleavage of epoxy and C-C bonds. Interestingly, the damaged GO and *in situ* formed GO fragments can further enhance decomposition of H_2_O_2_ to produce HO•. Different from other catalytic processes, the GO-TCBQ metal-free catalysis process can be enhanced by GO itself, producing more HO•, and uniform USGO also can be generated. Thus, the metal free catalysis will be considered a fabrication method for uniform USGO, and may be extended to other fields including detoxifying organic pollutants and the application as disinfectants.

Graphene oxide (GO), a highly oxidized form of graphene, has attracted widespread interests due to its unique physical and chemical properties^1^,^2^,^3^. Recently, research on GO-based metal-free catalyst is rapidly increasing^4^,^5^,^6^,^7^,^8^. In most cases, metal-based catalysts are hard to remove metal ions from the product, which would have a dominant effect on their further applications^9^,^10^. Thus, GO as metal-free catalyst has drawn more and more attention^10^,^11^,^12^.

As known, Fenton reaction is a common method for generating hydroxyl radical (HO•) via decomposition of hydrogen peroxide (H_2_O_2_) by metal-based catalysts^13^,^14^,^15^. Interestingly, GO itself can act as catalyst and react with H_2_O_2_ due to intrinsic peroxidase catalytic activity^16^,^17^,^18^,^19^. Sun H. *et al*. indicated graphene quantum dots (GQDs) could catalyze the decomposition of H_2_O_2_, generating HO• to improve the antibacterial performance of H_2_O_2_ effectively^20^. However, peroxidase catalytic activity of GO is usually sensitive to H_2_O_2_ with a very low concentration, resulting in the limited catalytic ability during the degradation of organic pollutants^15^. Therefore, it is a big challenge to improve catalytic activity of GO metal-free catalyst for decomposition of H_2_O_2_ to produce HO•.

On the other hand, hydroxyl radical has been reported to oxidize and cut GO into zero dimensional ultra-small graphene oxide (USGO) or GQDs^21^,^22^,^23^,^24^, which have various potential applications in the fields of optoelectronics^25^, catalysis^26^ and biology and medicine^27^. Now, various methods have been applied for the cleavage of GO into USGO or GQDs. However, most those methods require harsh conditions^28^,^29^,^30^,^31^ or complicated processes^28^.

Herein, for the first time, we report chloro-benquinone (TCBQ) modified on GO as metal-free catalyst, with a Janus effect for strong promotion of HO• and formation of USGO with uniform size and shape (Fig. 1). Based on density functional theory (DFT) and metal-free catalysis experiments, GO can effectively accelerate nucleophilic reaction between TCBQ modified GO (GO-TCBQ) and H_2_O_2_ to yield HO• due to promotion of electrons transfer from H_2_O_2_ to TCBQ. More importantly, the generated HO• can oxidize and cut GO nanosheets into USGO with uniform size and shape. Interestingly, the GO fragments itself can further enhance catalytic decomposition of H_2_O_2_ to produce more HO•.

## Results and Discussion

Zhu *et al*.^32^,^33^ demonstrated that H_2_O_2_ as a nucleophile could nucleophilic attack TCBQ, forming a trichlorohydroperoxyl-1,4-benzoquinone intermediate, which decomposes homolytically to produce HO•. Previous study also indicated that GO possesses intrinsic peroxidase-like activity to catalyze the reduction of H_2_O_2_^16^,^20^. In this study, we used TCBQ modified on the GO surface as metal-free catalyst to enhance H_2_O_2_ decomposition, which was named as GO-TCBQ. Some Details about characterization and optimization of GO-TCBQ were shown in Supplementary Material (Fig. S1 and S2).

Figure 2 shows the production of hydroxyl radical in the metal-free catalysis system. GO-TCBQ can significant enhance the decomposition of H_2_O_2_ to generate hydroxyl radical. As illustrated in the Fig. 2a, after dosing GO-TCBQ in the H_2_O_2_ solution, hydroxyl radical can be detected by electron spin resonance (ESR). As there was no metal catalyst in the system, hydroxyl radical is related to the interaction between GO-TCBQ and H_2_O_2_. Figure 2b also shows the quantities of hydroxyl radical in different H_2_O_2_ system. It is consistent with previously reported that TCBQ can decompose H_2_O_2_ to produce HO•^32^,^34^. In compared with TCBQ and GO, the production of hydroxyl radical by GO-TCBQ after 2 h reaction is enhanced 3 times and 13 times higher, respectively. Due to the non-selectivity of hydroxyl radical to electron-rich organic pollutants, metal-free catalyst GO-TCBQ accelerates the removal of organic pollutants with the presence of H_2_O_2_ as Fig. 2c indicated. Only GO does little effect on the decomposition of H_2_O_2_, so the removal of phenol in the GO-H_2_O_2_ system is almost the same as only H_2_O_2_ system. Furthermore, the production of hydroxyl radical by GO-TCBQ metal-free catalyst keeps increasing with reaction time. The yield of hydroxyl radical increases fast at the beginning, but the increase slows down after 5 hours. After 24 h reaction, the amount of hydroxyl radical shows 2 times higher than radical produced by 2 h reaction.

To explore the promotion of hydroxyl radical by GO-TCBQ, related theoretical calculations based on DFT were analyzed. Highest occupied molecular orbital (HOMO) and lowest unoccupied molecular orbital (LUMO) determine the way the molecule interacts with other species^35^. Figure 3 and Table 1 indicate the optimized configurations, and the HOMO/LUMO energy levels of the TCBQ, GO GO-TCBQ obtained by DFT calculations. The LUMO energy level of the GO-TCBQ (−4.13 eV) is lower than that of TCBQ (−3.90 eV) suggests that LUMO level becomes lower via TCBQ modified on GO surface (Fig. 3b). Thus, GO-TCBQ is more prone to obtain electron than TCBQ. It is consistent with a previously suggested that lower LUMO level benefited the injection of electron^36^. Figure 3c shows the nucleophilic reaction between TCBQ and H_2_O_2_. The chemical reactivity of molecular systems is associated closely with the energy difference E_g_ between HOMO and LUMO^37^,^38^, which is calculated for TCBQ and GO-TCBQ system. The E_g_ between the HOMO level of H_2_O_2_ and the LUMO level of TCBQ is 2.28 eV (52.4 kcal/mol), indicating electron transfer from the HOMO of H_2_O_2_ to the LUMO of TCBQ. The calculated E_g_ of GO-TCBQ is 2.05 eV (47.2 kcal/mol), which is considerably lower than that of TCBQ differences of E_g_ between GO-TCBQ and GO can well explain why the production of hydroxyl radical of H_2_O_2_ is enhanced by GO-TCBQ. Due to the promotion of electron transfer, GO-TCBQ is more prone to be attacked by H_2_O_2_. The Fig. 3a indicates that the LUMO isosurface of GO-TCBQ is very similar with that of TCBQ. That means electron also transfers from H_2_O_2_ to TCBQ in the GO-TCBQ/H_2_O_2_ system. Thus, GO promotes nucleophilic reaction through accelerating electron transfer from H_2_O_2_ to GO-TCBQ.

In the GO-TCBQ metal-free system mentioned above, hydroxyl radical can produce gradually through nucleophilic reaction. However, hydroxyl radical as electrophile can oxidize and decompose GO-TCBQ. As shown in Fig. 3a, the HOMO isosurface of GO-TCBQ is very similar with that of GO, indicating electron transport from GO to hydroxyl radical in this system. Thus, GO is more prone to electrophilic attack by hydroxyl radical. So, the morphology changes of GO during metal-free catalysis were conducted by transmission electron microscopy (TEM) and high-resolution transmission electron microscopy (HRTEM). TEM images in Fig. 4a–e indicate a process of USGO generation in the metal-free system of GO-TCBQ and H_2_O_2_. Firstly, a single-layer GO (Fig. 4a) gradually changes after 2h-reaction (Fig. 4b). The surface of GO sheet begins to damage, and some small holes can be found on the GO layer. As mentioned above, H_2_O_2_ can attack GO-TCBQ to produce *in situ* hydroxyl radical via nucleophilic reaction. The hydroxyl radical generated from GO could attack GO directly. Thus, when the solution is filtrated after 2h-reacion of metal-free catalysis (Fig. 4c), the change of GO fragments in the filtrate is significant. Most the structure of GO layer is damaged by the *in situ* hydroxyl radical. The visible holes or defect sites are generated initially by the attack of hydroxyl radical followed by progressive attraction of more hydroxyl radical to destroy the carbon-carbon bond around initial defect sites^39^. Thus, it is surprising that USGO with uniform size formed and GO layer disappears with the filtrate of 2h-reaction sample after 12h-reaction (Fig. 4d). This could be attributed to the oxidation of hydroxyl radical generated from GO-TCBQ. Then, GO is attacked and decomposed *in situ* by hydroxyl radical, where TCBQ is modified. Thus, USGO can be generated uniformly. After a longer time reaction (24–48 h), the USGO particles can be further decomposed into smaller ones (Fig. 4e,f). HRTEM spectra in Fig. 4f,h show the USGO particles are uniform, with a size distribution of 2–4 nm. The average diameter of the USGO is 3.61 nm. As Fig. 4g shown, the marked lattice fringe spacing of the USGO is 0.34 nm, corresponding to (002) crystal phase of graphite^21^. The size of USGO obtained during GO-TCBQ metal-free catalysis is similar that prepared in other metal catalysis system^22^,^23^. The particle sizes of USGO gradually decrease with reaction time (Fig. 4i), which is consistent with the production of hydroxyl radical.

In order to examine the chemical changes of GO during metal-free catalysis, we employed X-ray photoelectron spectroscopy (XPS), fourier transform infrared spectroscopy (FTIR), Raman, X-ray diffraction (XRD) and fluorescence spectroscopy to characterize the GO samples with different reaction time. The XPS ^1^C spectra in Fig. 5a–d indicate the distribution of functional groups on the surface of GO samples. The major peaks at 284.7, 285.3, 286.8 and 288.8 eV are corresponded to aromatics (C=C/C–C), hydroxyl and epoxy (C–O), carbonyl (C=O), and carboxyl (C(O)O)groups, respectively. Table 2 shows the corresponding functional group contents of C1s spectra in XPS analysis. As the reaction time of metal-free catalysis increased, carbonyl groups (peak at 286.8 eV) largely decrease, from 60.3% to 35.4%. However, carboxyl (288.8 eV), hydroxyl and epoxy (285.3 eV) and aromatics (284.7 eV) groups increase. The C-O group increased from 6.04% to 18.0%, and C(O)O group increased from 0.73% to 3.53%. This indicated carbonyl groups were oxidized into C-O and C(O)O groups during metal-free catalysis. The carbonyl groups on the surface of GO-TCBQ may be easily attacked and oxidized by the *in situ* hydroxyl radical into the USGO containing carboxyl groups. The increase of hydroxyl groups may be due to the replacement by chlorine atom from GO-TCBQ through nuclephilic substitution of TCBQ and H_2_O_2_.

The functional groups of the GO samples also are determined by the FT-IR spectra shown in Fig. 6a. The peaks at 1630 cm^−1^ is assigned as C=O stretching of carbonyl groups, 1575 cm^−1^ represents C=C stretching of aromatic rings, 1380 cm^−1^ is due to C-H deformation of CH_2_ and CH_3_ groups, the absorbance at 1105 cm^−1^ is attributed to O-H deformation of COOH, and the band at 1040 cm^−1^ is characteristic of aromatic C-O-C stretching of aryl ethers (epoxy groups). FT-IR results clearly show a reduction in carbonyl (1630 cm^−1^) responses, and an increase in O-H deformation of COOH (1105 cm^−1^) response. It is consistent with the XPS results mentioned above. The C=O groups of GO-TCBQ may be easily attacked and oxidized by hydroxyl radical. Furthermore, aromatic C-O-C groups (1040 cm^−1^) disappear from the GO samples after 2h-reaction of metal-free catalysis. It should be noted that epoxy (aromatic C-O-C) groups can serve as chemically reactive sites for the rupture of the underlying C-C bonds. Thus, *in situ* hydroxyl radical mainly attacks epoxy groups in the GO samples. On the other hand, CH_2_/CH_3_ groups (1380 cm^−1^) in the Fig. 6a increase after 24h-reaction of metal-free catalysis, indicating further decomposition of GO nanosheets by hydroxyl radical through the cleavage of C-C bonds.

The XRD is used to determine the crystal structure of GO and USGO. The Bragg’s equation is applied to evaluate the distance between graphene layers (nλ = 2dsinθ, where λ is the wavelength, d is the distance between crystal planes, θ is the angle of the diffracted wave, and n is an integer known as the order of the diffracted beam). As shown in Fig. 6b, the original GO-TCBQ has a diffraction peak at 10.76°, and the interlayer spacing is 0.8 nm. The XRD peak shifts to a lower degree after 2h-reaction, and further shifts after filtration. The interlayer distances of GO increase to 0.82 nm and 1.32 nm, respectively. This result could be attributed to the oxygen-containing groups introduced GO during metal-free catalysis, which enhances the interlayer spacing. After 24h-reaction, the XRD peak of USGO shifts to 10.79°, and the interlayer spacing of USGO is 0.78 nm, indicating the decrease of interlayer distance. This may be due to carbonyl groups on the surface of GO attacked by hydroxyl radical, which is according with the XPS and FTIR results mentioned above. Other research indicated oxygen-containing functional groups on the surface of GO affected surface characteristics of GO^40^,^41^, especially for interlayer spacing of GO^42^. In this study, the content of oxygen-containing groups decreased from 67.07% to 56.93% during oxidation process. Thus, the content of oxygen-containing groups was consistent with the decrease of interlayer distance.

The major Raman peaks in GO samples are the D and G band at around 1352 cm^−1^ and 1600 cm^−1^, respectively (Fig. 6c). The intensity ratio of disorder D and crystalline G band (I_D_/I_G_) reflects the degree of defects in the GO. The GO-TCBQ sample is observed with an intensity ratio I_D_/I_G_ at 0.88, after 2h-reaction the intensity nearly does not change. But the defect intensity increases to 1.00 after filtration, which is an indication of oxygen-containing groups increasing in the GO fragments. Then I_D_/I_G_ decreases to 0.76 after 24h-reaction, indicates the USGO with a higher sp^2^ crystalline structure are gradually formed. Due to oxygen-containing groups of GO attacked and cut by hydroxyl radical, oxygen groups gradually move to the edge of USGO, which is similar with XRD results.

Fluorescence spectra are carried to determine the emission of the GO samples at a fixed excitation of 335 nm. As shown in Fig. 6d, both GO and TCBQ samples show weak peaks with an emission of 380 nm. After 2 h metal-free catalysis, the strong fluorescence peak could be observed at an emission of 426 nm. Compared with original GO, the fluorescence intensity of the GO samples increased 7 times after 2h-reaction. After 24 h, USGO shows a strong peak at an emission wavelength of 438 nm. With a red shift of the emission wavelength, the fluorescence intensity also reaches the maximum value, increasing more than 10 times. The fluorescence properties of USGO should be attributed to the uniform nano-size and the surface state of the sp^2^ clusters. It is consistent with previous studies that the blue emission of GQDs is due to electron hole recombination or quantum size effect (intrinsic state emission)^31^,^43^,^44^. Chemical functionalities and defects could cause red-shift of the emission peaks^45^. Thus, the reason of red-shift may be attributed to the increase in C-O and C(O)O groups during GO cutting process (Table 2).

**Figure 1:**
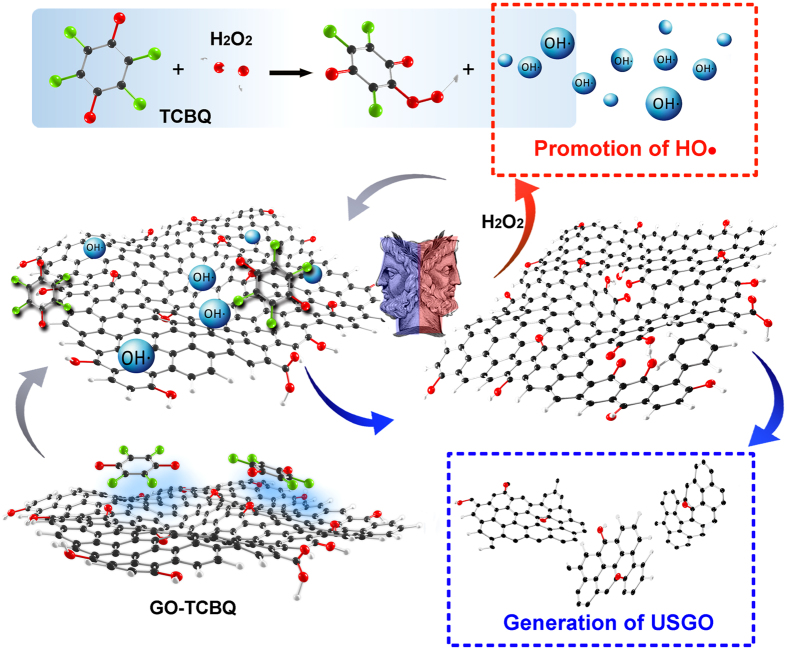
Schematic representation of the Janus effect mechanism of metal-free catalysis. (TCBQ modified on GO as metal-free catalyst is more prone to be nucleophilic attacked by H_2_O_2_ to yield HO• via electron transfer acceleration; on the other hand, the generated HO• can cut GO nanosheets into uniform USGO. GO fragments also further enhance decomposition of H_2_O_2_ to produce more HO•).

Based on the data and analysis presented above, a mechanism of uniform USGO formation during gentle metal-free catalysis is proposed, as detailed in Fig. 1. There is a Janus effect of process for promotion of hydroxyl radical and USGO generation.

On one hand, the production of hydroxyl radical is significantly enhanced in a gentle metal-free catalysis system. The nucleophilic reaction between TCBQ and H_2_O_2_ is promoted via TCBQ modified on the GO surface. GO-TCBQ is more prone to be attacked by H_2_O_2_ to yield HO• through the acceleration of electron transfer. On the other hand, the GO nanosheets are further cut into uniform USGO through metal-free catalysis. Firstly, the hydroxyl radical *in situ* produced from the GO-TCBQ can directly oxidize the surface of the GO. As discussed above, epoxy (aromatic C-O-C) and carbonyl groups as chemically reactive sites on the surface of GO is easily attacked by hydroxyl radical into carboxyl groups. Therefore, the structure of GO layer is damaged by the *in situ* hydroxyl radical through cleavage of epoxy C-O-C bonds. This is supported by TEM results (Fig. 4b), which clearly shows the small holes and damage on the surface of GO sheet after 2h-reaction of metal-free catalysis. Epoxy groups on the plane of GO were oxidized, resulting in the cutting of GO, which is consistent with previously reported results.

**Figure 2:**
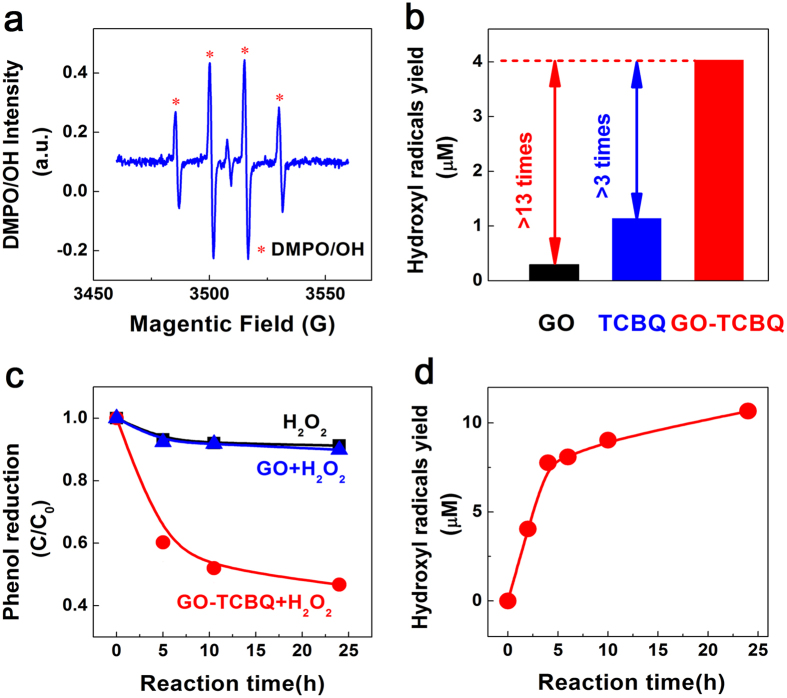
Generation of hydroxyl radical by different metal-free catalysts. (GO = 0.15 mg/mL, TCBQ/GO = 0.3, H_2_O_2_ = 2 mM, ultrasonic time = 1 h, pH = 7.0). (**a**) ESR spectrum of GO-TCBQ with H_2_O_2_ after 1 min reaction. (**b**) Quantitative yield of hydroxyl radicals by LC-MS using salicylic acid method in H_2_O_2_ system (H_2_O_2_ = 2 mM, 2,3-DHBA as the quantitative product by hydroxyl radical). (**c**) Removal of phenol with reaction time by metal-free catalysts (original phenol concentration = 10 mg/L). (**d**) Quantitative yield of hydroxyl radicals with reaction time by GO-TCBQ metal-free catalyst (salicylic acid method).

**Figure 3:**
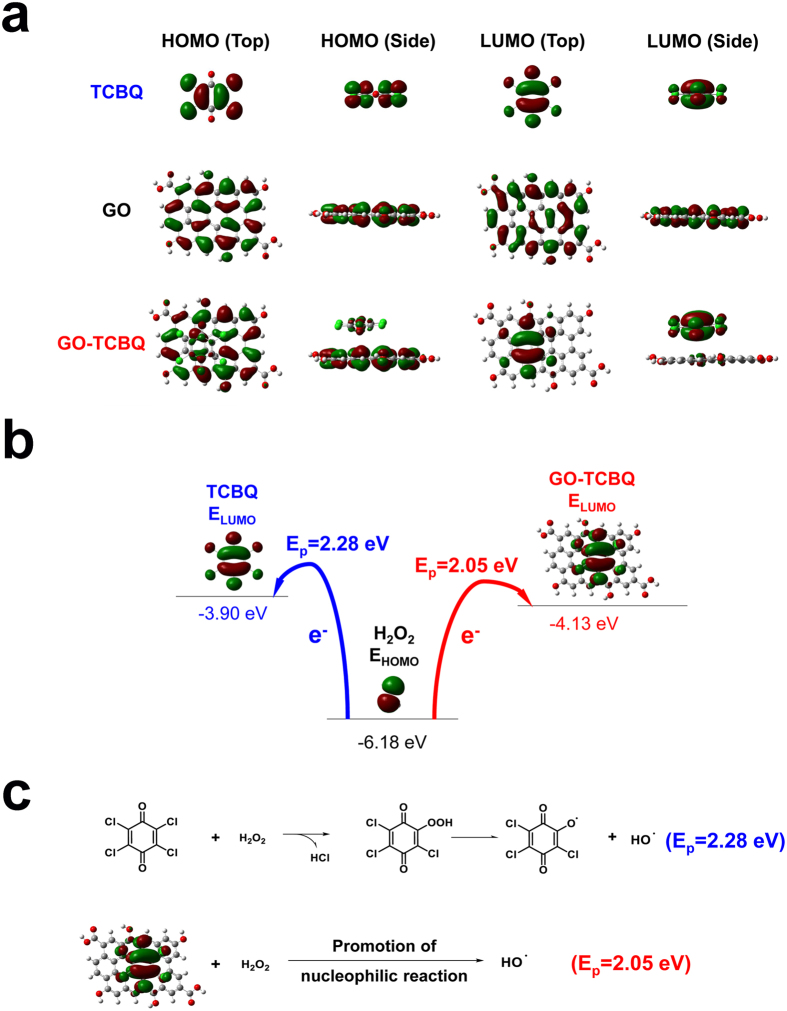
Theoretical calculations using DFT at 6–31 G (d,p)-B3LYP. (**a**) The HOMO and LUMO density distribution for optimized configurations. (**b**) Comparison of energy levels and calculated HOMO-LUMO energy gaps for TCBQ and GO-TCBQ reaction system. (**c**) Schematic illustrations of nucleophilic reaction between TCBQ (GO-TCBQ) and H_2_O_2_.

**Figure 4:**
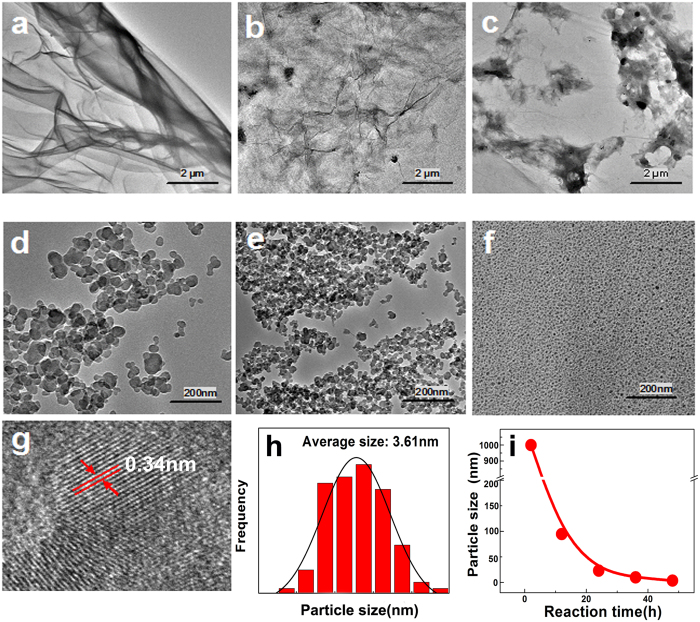
TEM and HRTEM images of GO-TCBQ decomposition process with reaction time in metal-free catalysis. (GO = 0.15 mg/mL, TCBQ/GO = 0.3, H_2_O_2_ = 2 mM, ultrasonic time = 1 h, pH = 7.0). (**a**) GO-TCBQ, (**b**) GO-TCBQ after 2h-reaction, (**c**) filtrate of GO-TCBQ after 2h-reaction, (**d**) filtrate of (**c**) after 12h-reaction, (**e**) filtrate of (**c**) after 24h-reaction, (**f**) filtrate of (**c**) after 48h-reaction, (**g**) fringe patterns of individual USGO, (**h**) particle size distributions of USGO, (**i**) particle sizes of USGO with reaction time.

**Figure 5:**
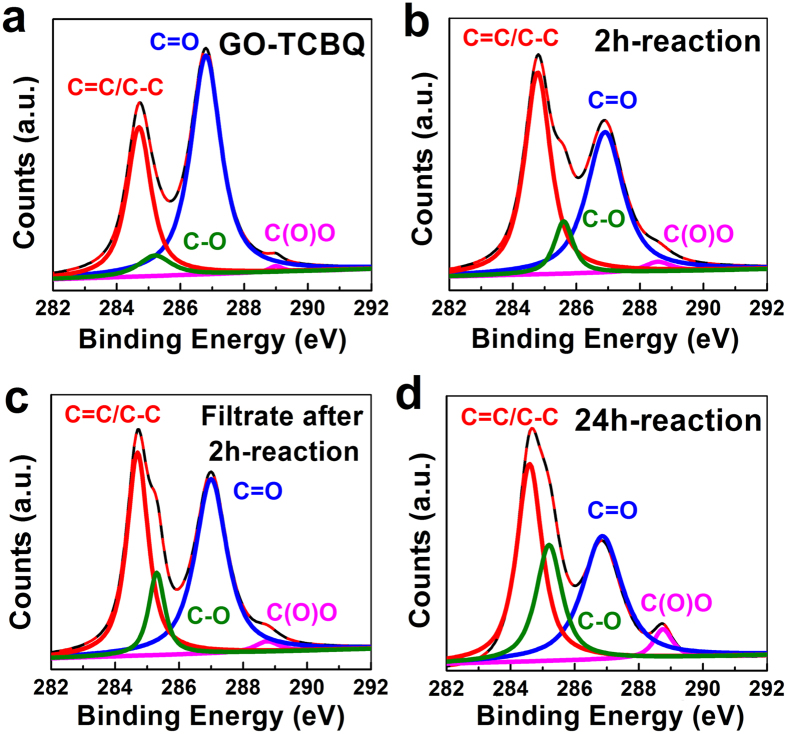
XPS ^1^C spectra of samples. (GO = 0.15 mg/mL, TCBQ/GO = 0.3, H_2_O_2_ = 2 mM, ultrasonic time = 1 h, pH = 7.0), (**a**) GO-TCBQ, (**b**) GO-TCBQ after 2h-reaction, (**c**) the filtrate of GO-TCBQ after 2h-reaction, (**d**) the filtrate of (**c**) after 24h-reaction.

**Figure 6:**
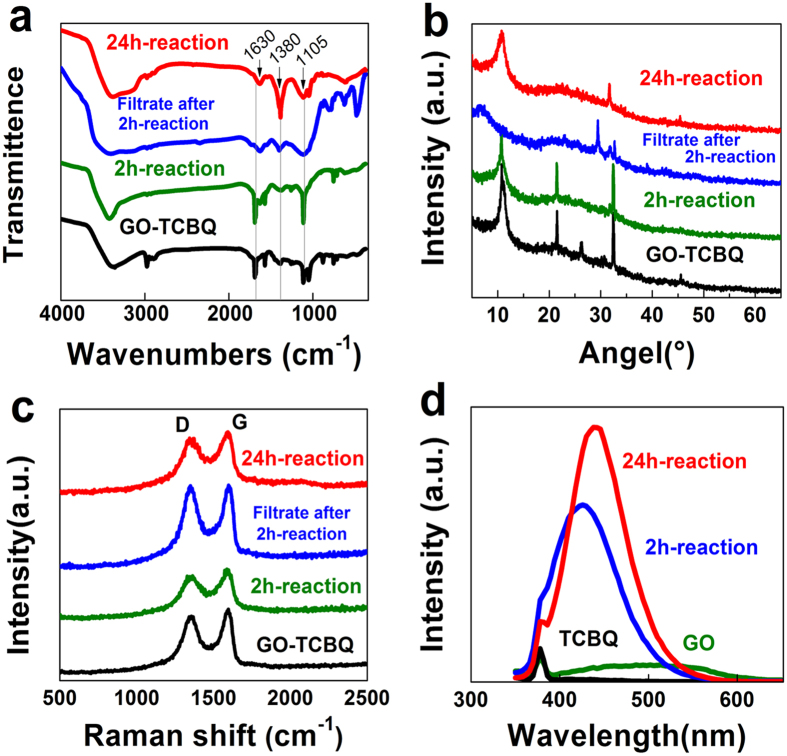
(**a**) FTIR, (**b**) XRD, (**c**) Raman and (**d**) fluorescence spectra (Ex = 335 nm) of different samples (GO = 0.15 mg/mL, TCBQ/GO = 0.3, H_2_O_2_ = 2 mM, ultrasonic time = 1 h, pH = 7.0).

Secondly, GO nanosheets are further cut into extra-small sheets also could be enhanced by GO itself. Due to the structural changes of TCBQ, the promotion of hydroxyl radical by TCBQ and H_2_O_2_ nucleophilic reaction would slow down after 5 h (Fig. 2d). However, As Fig. 4 shown, the decomposition of GO was still ongoing after 5 h reaction. Some investigations indicated that carboxyl-modified GO is shown to possess intrinsic peroxidase-like activity^16^. With smaller size, USGO possess higher peroxidase-like activity^17^ originates from their ability to catalyze the decomposition of H_2_O_2_ to generate hydroxyl radical^23^. In this study, the *in situ* formed GO fragments might further enhance the decomposition of H_2_O_2_ to produce more hydroxyl radical during 24 h or longer metal-free catalysis. Furthermore, it should be noted that a further cutting of GO nanosheets was by cleavage of C-C bonds. During the initial metal-free catalysis, GO is attacked by hydroxyl radical to form holes and defect sites, around where more hydroxyl radical can be progressive attracted to destroy the carbon-carbon bond^18^,^46^.

Therefore, GO-TCBQ, as metal-free catalyst, can produce hydroxyl radical with the presence of H_2_O_2_, to enhance degradation of organic pollutants. Besides environmental application in detoxifying pollutants, GO-TCBQ also had other application potentials. It has been found that the GO-TCBQ with ability to catalyze the decomposition of H_2_O_2_, generating HO•. Thus, GO-TCBQ can be used for H_2_O_2_ detection or wound disinfection.

## Conclusion

In summary, a Janus effect of gentle metal-free catalysis facilitated production of hydroxyl radical and the fabrication of the uniform USGO. The production of hydroxyl radical is significantly enhanced by GO-TCBQ metal-free catalyst. GO-TCBQ is more prone to be nucleophilic attacked by H_2_O_2_ to yield HO• via electron transfer acceleration. On the other hand, the GO sheets are further cut into uniform USGO by HO•. The damaged GO itself also can decompose of H_2_O_2_ to produce HO• to cut GO into USGO. Different from other catalytic processes, the metal-free catalysis can be enhanced by GO itself, producing more hydroxyl radical, and uniform USGO also can be generated. The metal-free catalysis will be considered a fabrication method for uniform USGO, and may be extended to other fields including detoxifying organic pollutants and the application as disinfectants.

## Methods

### Preparation of GO and GO-TCBQ

Graphite oxide was prepared by a modified Hummers method^47^,^48^. The obtained graphite oxide powder was then redispersed in water to yield a yellow-brown suspension. 1 mg/mL GO aqueous suspension was dropwise added into TCBQ solution and then sonicated under ambient condition (300 W) for 60 min. The obtained TCBQ modified GO was used to the preparation of USGO. The basic system consisted of 0.2 mM TCBQ, 0.15 mg/mL GO, 2 mM H_2_O_2_, in 0.1 M phosphate buffer (pH 7.4) and was magnetic stirred at 25 °C in water bath. After 2 hours, the solution was filtrated by 0.22 μm membrane, and the filtrate reacted continuously until 48 hours. The colloidal solution was further dialyzed in a dialysis bag (retained molecular weight: 3500 Da) for 2 days and USGO were obtained.

### Metal-free catalyzed process of GO-TCBQ and H_2_O_2_

Metal-free catalyzed process was carried out in a dark reactor. Salicylic acid method was used to quantify the hydroxyl radical generated by hydrogen peroxide with TCBQ modified GO by ESI-TOF-MS methods. Typically, the basic system initially consisted of TCBQ modified GO and salicylic acid. The pH was adjusted to 7.4 by using PBS solution before reaction. After H_2_O_2_ was added, the system was magnetic stirred at 25 °C in water bath. The product was filtrated by 0.22 μm membrane filter immediately after the reaction for 2 h. LC-MS spectra were analyzed by UPLC H-Class/xevo G2-S TOF (Waters, USA) with a mass range of *m/z* 50–10000 and resolution ≥40. The area of the hydroxylation product (DHBA, *m/z* 153.02) was used to represent the hydroxyl radical yield.

The degradation experiment was carried out at 25 °C in water bath reactor at optimum conditions, containing 100 mL solution with 1 mM of phenol. The samples were taken at certain intervals and analyzed with high performance liquid chromatography (HPLC, Agilent Technologies Series 1200), using a mixture of methanol and water (30/70%, v/v) containing 10 mM/L H_3_PO_4_ as the mobile phase. The separation was performed using ZORBAX SB-C18 (3.5 μm, 2.1 × 150 mm) reversed-phase column at 35 °C with a flow rate of 0.25 mL/min. Sample was analyzed by a UV detector with a wavelength of 210 nm.

### Analysis method and characterization

In ESR study, the basic system consisted of halogenated quinones dissolved in acetonitrile (5%), H_2_O_2_, and the spin-trapping agent 5,5-Dimethyl-1-Pyrroline-N-Oxide (DMPO) (100 mM), in 100 mM phosphate buffer (pH 7.4) at room temperature. ESR spectra were recorded 1 min after the interactions at room temperature under normal room-lighting conditions on a Bruker (Billerica, MA) AE500 spectrometer operating at 9.8 GHz and a cavity equipped with a Bruker Aquax liquid sample cell. Typical spectrometer parameters were scan range, 100 G; field set, 3470 G; time constant, 200 ms; scan time, 100 s.

The morphology structure of the obtained material was characterized by using TEM (JEOLJEM-100CXII, Japan) and HRTEM (JEM-2100F, Japan) at an accelerating voltage of 300 kV. The FTIR spectra of the samples were recorded on a Spectra GX spectrometer (PerkinElmer, USA) operating under the transmittance mode. The FTIR spectra were acquired in the wavenumber range of 400–4000 cm^−1^ at the resolution of 1 cm^−1^. The XRD pattern was recorded on PANalytical X-ray diffraction system (Empyrean, Netherlands). XPS data was obtained on an electron spectrometer (ESCALab 250Xi, VG Scientific, Britain) using Al Kα radiation at the power of 300 W. Survey and multi-region spectra were recorded at C1s and O1s photoelectron peaks. The Raman spectra were obtained on a LabRAM HR800 Raman spectrometer (Horiba Jobin Yvon, France) with an excitation wavelength of 514 nm. The fluorescence spectra were recorded using a Hitachi F-4600 fluorescence spectrometer (Hitachi High-Technologies, Tokyo, Japan).

### Theoretical calculations details

The calculations were performed on a Intel Xeon E3–1225v5 server with 8 G memory using Gaussian 03 W^49^ program package. The input geometry of the GO, TCBQ and GO-TCBQ in the ground state was fully optimized at DFT/B3LYP using 6–311 G basis set (Details are shown in SI). All the calculations, HOMO and LUMO energy levels were performed by using GaussView 4.0 molecular visualization program Package^50^.

## Additional Information

**How to cite this article:** Zhao, H. *et al*. Chloro-benquinone Modified on Graphene Oxide as Metal-free Catalyst: Strong Promotion of Hydroxyl Radical and Generation of Ultra-Small Graphene Oxide. *Sci. Rep.* **7**, 42643; doi: 10.1038/srep42643 (2017).

**Publisher's note:** Springer Nature remains neutral with regard to jurisdictional claims in published maps and institutional affiliations.

## Supplementary Material

Supplementary Information

## Figures and Tables

**Table 1:** The HOMO energies (E_HOMO_), LUMO energies (E_LUMO_) and HOMO/LUMO energy gap (E_g_) for different systems.

System	E_HOMO_ (eV)	E_LUMO_ (eV)	E_g_ (eV)
TCBQ	−7.36	−3.90	3.45
GO	−4.70	−3.13	1.57
GO-TCBQ	−4.82	−4.13	0.69
H_2_O_2_	−6.18	0.36	6.54
TCBQ/H_2_O_2_	−6.18	−3.90	2.28
GO/H_2_O_2_	−6.18	−3.13	3.05
GO-TCBQ/H_2_O_2_	−6.18	−4.13	2.05

**Table 2:** Functional group contents obtained from the peak area ratios of C1s spectra in XPS analysis.

Samples	C=/C-C	C-O	C=O	C(O)O
BE(eV)	284.7	285.3	286.8	288.8
GO-TCBQ	32.39%	6.04%	60.3%	0.73%
2h-reaction	54.9%	8.45%	44.2%	2.25%
Filtrate after 2h-reaction	24.26%	11.6%	48.1%	2.22%
24h-reaction	56.93%	18.0%	35.4%	3.53%
