# Supplementary material for: Chloro-benquinone Modified on Graphene Oxide as Metal-free Catalyst: Strong Promotion of Hydroxyl Radical and Generation of Ultra-Small Graphene Oxide
Source: Sci Rep. 2017 Mar 28;7:42643. doi: 10.1038/srep42643 (PMC5368711; doi:10.1038/srep42643)
Supplement: Supplementary Information [file srep42643-s1.pdf]

# Supplementary Material

# Chloro-benquinone Modified on Graphene Oxide as Metal-free Catalyst: Strong Promotion of Hydroxyl Radical and Generation of Ultra-Small Graphene Oxide

**He Zhao[1], Juehua Wang[1], Di Zhang[1], Qin Dai[1], Qingzhen Han[2], Penghui Du[1], Chenming Liu[1], Yongbing Xie[1], Hongbin Cao[1,*], Zhuangjun Fan[3,*]**

[1] Beijing Engineering Research Center of Process Pollution Control, Division of Environment Technology and Engineering, Institute of Process Engineering, Chinese Academy of Sciences, Beijing 100190, China
[2] State Key Laboratory of Multiphase Complex System, Institute of Process Engineering, Chinese Academy of Sciences, Beijing 100190, China
[3] Key Laboratory of Superlight Materials and Surface Technology, Ministry of Education, College of Material Science and Chemical Engineering, Harbin Engineering University, Harbin 150001, Heilongjiang, China

*Corresponding Authors: hbcao@ipe.ac.cn (H.Cao), fanzhj666@163.com (Z. Fan).

## I. FT-IR characterization of GO and GO-TCBQ

GO-TCBQ was prepared via TCBQ modified on GO. The FT-IR characterization of GO and GO-TCBQ is shown in Fig. S1. Comparing with GO, the FT-IR spectrum of GO-TCBQ clearly show a new peak at 2975 $cm^{-1}$, which represents unsaturated C-H. Other peaks also increased significantly in the FTIR results of GO-TCBQ, including C=O (1691 $cm^{-1}$), C=C (1571 $cm^{-1}$) and O-H (1114 $cm^{-1}$) and C-O-C (1049 $cm^{-1}$) groups. TCBQ had similar peaks of C=O, C=C and Arc-H (Fig. S2), indicating these groups from TCBQ. Additionally, it should be noted that increased O-H and C-O-C groups may origin from carbonyl of TCBQ. Thus, TCBQ was grafted on GO through C-O-C or O-H bonding.

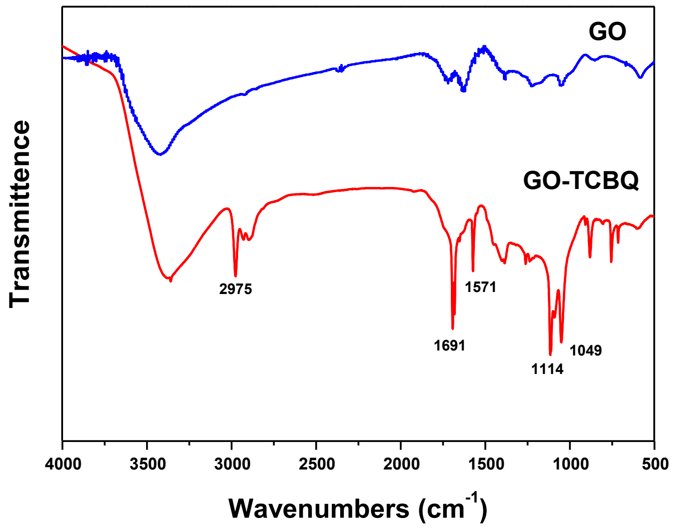


**Fig. S1.** FTIR spectra of GO and GO-TCBQ samples. (GO=0.15 mg/mL, TCBQ/GO=0.3, ultrasonic time=1h, pH=7.0).

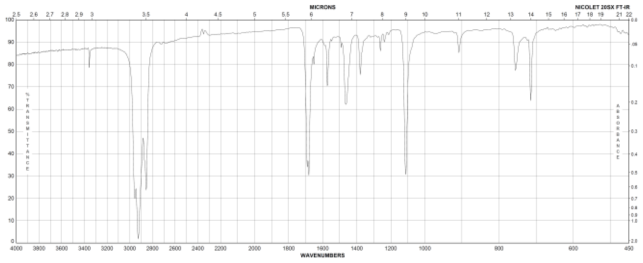

**Fig. S2.** FTIR spectrum of TCBQ (http://www.sigmaaldrich.com/catalog/product/aldrich/232017?lang=zh&region=CN)

## II. Influences the hydroxyl radical production.

In the GO-TCBQ metal free system, the decomposition of $H_2O_2$ and the production of hydroxyl radical were strongly dependent on pH, reactant ratios, and reaction time. The influence of oxidation states of graphene, pH values, GO/TCBQ ratios, ultrasonic time and $H_2O_2$/GO ratios were optimized in the Fig. S1. As illustrated in Fig. S1a, GO-TCBQ metal free system catalyzed to produce more hydroxyl radical than rGO (rGO-TCBQ) system. Fig.S1b shows hydroxyl radical is produced at pH 7 to the most, indicates

neutral pH condition is optimal for the metal free catalysis. As shown in Fig. S1c,d, the optimized ratio of GO/TCBQ and ultrasonic time was 0.3 and 1h in the metal free system, respectively. Furthermore, a filter process and longer reaction time will benefit the formation of hydroxyl radical during metal free catalysis. Fig. S1e indicates the ratios of TCBQ/$H_2O_2$ can affect the production of hydroxyl radical. When the ratio of TCBQ/$H_2O_2$ is 1/10, most hydroxyl radical is produced. During 0-5h reaction, hydroxyl radical sharply increased, and the yields reached maximum at 24h.

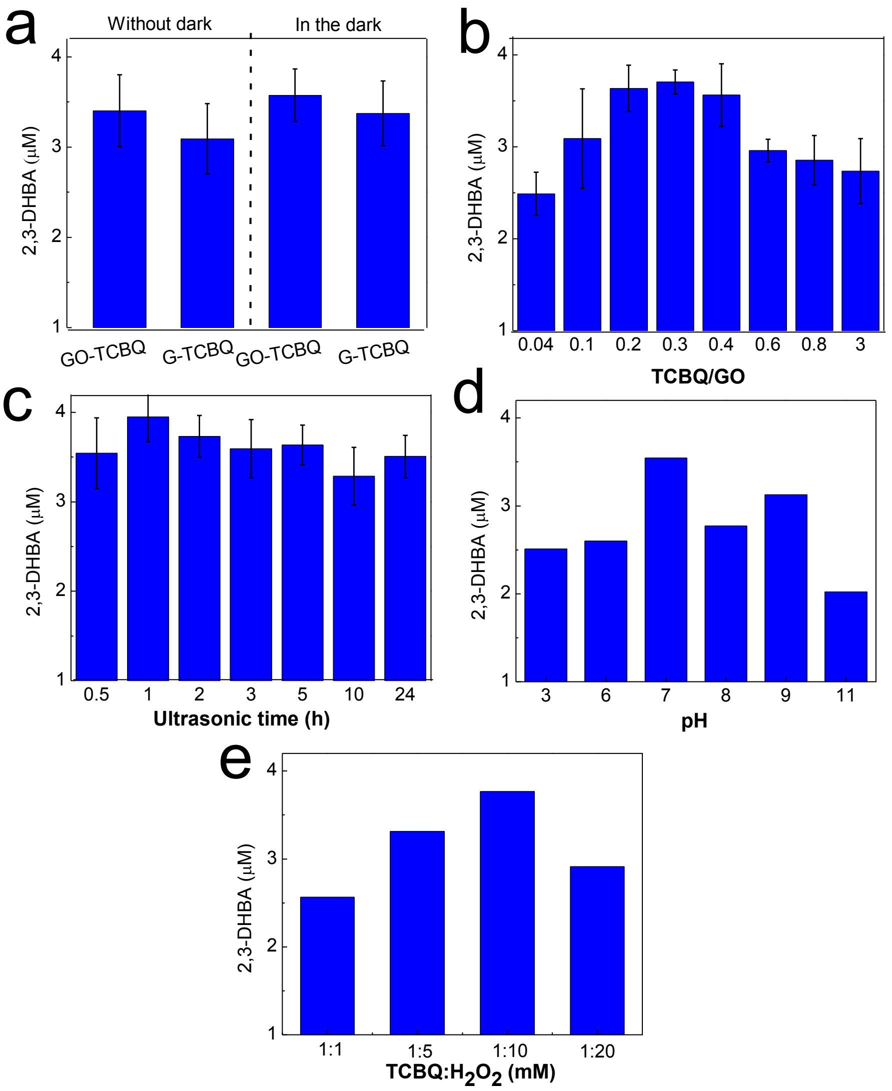


**Fig. S2.** Optimization of hydroxyl radical production in metal free catalytic reaction (GO or rGO=0.15mg/mL, TCBQ/GO=0.3, $H_2O_2$=2mM, ultrasonic time=1h, pH=7.0, 2h), (a) effect of Graphene and light, (b) effect of TCBQ/GO ratio (w/w), (c) effect of ultrasonic time, (d) effect of pH, (e) effect of $H_2O_2$/TCBQ ratio (4h).

**III. Molecular information of GO materials.**

The molecular formulas of GO-TCBQ, GO and TCBQ are $C_{46}H_{16}Cl_4O_{10}$, $C_{40}H_{16}O_8$ and $C_6Cl_4O_2$, respectively. The information is as followed.

**Table S1. Standard orientation of GO-TCBQ.**

| Center Number | Atomic Number | Atomic Type | Coordinates (Angstroms) X | Y | Z |
|---|---|---|---|---|---|
| 1 | 6 | 0 | -0.348540 | -1.770753 | 1.480643 |
| 2 | 6 | 0 | -1.748959 | -1.230803 | 1.488947 |
| 3 | 6 | 0 | -1.990529 | 0.096540 | 1.588215 |
| 4 | 6 | 0 | -0.868850 | 1.088174 | 1.694454 |
| 5 | 6 | 0 | 0.531569 | 0.548226 | 1.686142 |
| 6 | 6 | 0 | 0.773139 | -0.779118 | 1.586874 |
| 7 | 8 | 0 | -0.131674 | -2.962357 | 1.391531 |
| 8 | 8 | 0 | -1.085716 | 2.279777 | 1.783579 |
| 9 | 17 | 0 | 2.357325 | -1.448949 | 1.572794 |
| 10 | 17 | 0 | -2.997020 | -2.407864 | 1.364899 |
| 11 | 17 | 0 | -3.574715 | 0.766372 | 1.602288 |
| 12 | 17 | 0 | 1.779631 | 1.725287 | 1.810184 |
| 13 | 6 | 0 | 5.929862 | -1.386611 | -0.493276 |
| 14 | 6 | 0 | 5.580562 | -0.000506 | -0.466631 |
| 15 | 6 | 0 | 4.263522 | 0.402777 | -0.488433 |
| 16 | 6 | 0 | 3.210963 | -0.589747 | -0.539439 |
| 17 | 6 | 0 | 3.574543 | -1.971911 | -0.565462 |
| 18 | 6 | 0 | 4.951407 | -2.353562 | -0.541541 |
| 19 | 6 | 0 | 1.845879 | -0.217218 | -0.563702 |
| 20 | 6 | 0 | 0.834442 | -1.207337 | -0.613598 |
| 21 | 6 | 0 | 1.173368 | -2.592267 | -0.640022 |
| 22 | 6 | 0 | 2.541022 | -2.941345 | -0.615210 |
| 23 | 6 | 0 | -0.563329 | -0.838771 | -0.638951 |
| 24 | 6 | 0 | -1.557359 | -1.823093 | -0.688161 |
| 25 | 6 | 0 | -1.186522 | -3.230518 | -0.714124 |
| 26 | 6 | 0 | 0.139950 | -3.584323 | -0.690288 |
| 27 | 6 | 0 | -2.948135 | -1.443027 | -0.713142 |

| | | | | | |
|---|---|---|---|---|---|
| 28 | 6 | 0 | -3.967331 | -2.437718 | -0.763050 |
| 29 | 6 | 0 | -3.540805 | -3.835196 | -0.787239 |
| 30 | 6 | 0 | -2.240833 | -4.226485 | -0.764902 |
| 31 | 6 | 0 | 3.894180 | 1.827783 | -0.461072 |
| 32 | 6 | 0 | 2.487894 | 2.187717 | -0.486961 |
| 33 | 6 | 0 | 1.479047 | 1.191559 | -0.537273 |
| 34 | 6 | 0 | 0.113186 | 1.557163 | -0.562048 |
| 35 | 6 | 0 | -0.926544 | 0.556179 | -0.613068 |
| 36 | 6 | 0 | -2.276839 | 0.924812 | -0.637466 |
| 37 | 6 | 0 | -3.317490 | -0.070531 | -0.688195 |
| 38 | 6 | 0 | -4.704730 | 0.296305 | -0.713566 |
| 39 | 6 | 0 | -5.670295 | -0.711642 | -0.762321 |
| 40 | 6 | 0 | -5.309353 | -2.063447 | -0.786895 |
| 41 | 6 | 0 | 4.850473 | 2.814106 | -0.411830 |
| 42 | 6 | 0 | 4.448332 | 4.187030 | -0.386857 |
| 43 | 6 | 0 | 3.133376 | 4.579817 | -0.409896 |
| 44 | 6 | 0 | 2.118771 | 3.569867 | -0.461063 |
| 45 | 6 | 0 | 0.754048 | 3.925332 | -0.486219 |
| 46 | 6 | 0 | -0.233625 | 2.943993 | -0.535735 |
| 47 | 6 | 0 | -1.644865 | 3.281442 | -0.562564 |
| 48 | 6 | 0 | -2.647005 | 2.323569 | -0.611691 |
| 49 | 6 | 0 | -4.044821 | 2.662958 | -0.638317 |
| 50 | 6 | 0 | -5.032734 | 1.721603 | -0.686528 |
| 51 | 8 | 0 | 5.503587 | 5.061643 | -0.338002 |
| 52 | 6 | 0 | -6.403369 | 2.278430 | -0.707410 |
| 53 | 8 | 0 | -4.594507 | -4.712759 | -0.835022 |
| 54 | 8 | 0 | -6.700091 | 3.458109 | -0.685123 |
| 55 | 8 | 0 | -7.421791 | 1.358690 | -0.756653 |
| 56 | 6 | 0 | 7.345407 | -1.801226 | -0.469375 |
| 57 | 8 | 0 | 7.817659 | -2.915558 | -0.486289 |
| 58 | 8 | 0 | 8.193871 | -0.713247 | -0.420960 |
| 59 | 8 | 0 | 2.966220 | -4.238875 | -0.637433 |
| 60 | 8 | 0 | -1.884261 | 4.621850 | -0.534900 |

| | | | | | |
|---|---|---|---|---|---|
| 61 | 1 | 0 | 6.400384 | 0.728803 | -0.428176 |
| 62 | 1 | 0 | 5.204062 | -3.422795 | -0.562416 |
| 63 | 1 | 0 | 0.436823 | -4.633141 | -0.709173 |
| 64 | 1 | 0 | -1.950681 | -5.271565 | -0.783710 |
| 65 | 1 | 0 | -6.737837 | -0.464568 | -0.782536 |
| 66 | 1 | 0 | -6.083869 | -2.835971 | -0.825147 |
| 67 | 1 | 0 | 5.920909 | 2.601451 | -0.390392 |
| 68 | 1 | 0 | 2.838981 | 5.623293 | -0.390725 |
| 69 | 1 | 0 | 0.465861 | 4.979369 | -0.466785 |
| 70 | 1 | 0 | -4.304659 | 3.732989 | -0.618301 |
| 71 | 1 | 0 | 5.206853 | 6.006787 | -0.321311 |
| 72 | 1 | 0 | -4.294155 | -5.657801 | -0.851087 |
| 73 | 1 | 0 | -8.317903 | 1.802878 | -0.768333 |
| 74 | 1 | 0 | 9.151137 | -0.987362 | -0.404637 |
| 75 | 1 | 0 | 2.198176 | -4.881419 | -0.672465 |
| 76 | 1 | 0 | -2.861685 | 4.839138 | -0.553953 |

**Table S2. Standard orientation of GO.**

| Center | Atomic | Atomic | Coordinates (Angstroms) | | |
|---|---|---|---|---|---|
| Number | Number | Type | X | Y | Z |
| 1 | 6 | 0 | 5.730499 | -1.349527 | -0.000103 |
| 2 | 6 | 0 | 5.347478 | 0.010213 | -0.000085 |
| 3 | 6 | 0 | 4.009815 | 0.390152 | -0.000043 |
| 4 | 6 | 0 | 2.999968 | -0.628936 | 0.000021 |
| 5 | 6 | 0 | 3.398253 | -2.009477 | -0.000038 |
| 6 | 6 | 0 | 4.771338 | -2.347042 | -0.000076 |
| 7 | 6 | 0 | 1.621377 | -0.293677 | -0.000121 |
| 8 | 6 | 0 | 0.645202 | -1.320379 | 0.000099 |
| 9 | 6 | 0 | 1.032306 | -2.709581 | 0.000029 |
| 10 | 6 | 0 | 2.404754 | -3.019332 | -0.000021 |
| 11 | 6 | 0 | -0.744035 | -0.989175 | -0.000078 |
| 12 | 6 | 0 | -1.729064 | -2.004457 | 0.000121 |

| | | | | | |
|---|---|---|---|---|---|
| 13 | 6 | 0 | -1.327481 | -3.390886 | 0.000129 |
| 14 | 6 | 0 | 0.020938 | -3.707710 | 0.000102 |
| 15 | 6 | 0 | -3.113827 | -1.669278 | 0.000067 |
| 16 | 6 | 0 | -4.099846 | -2.700504 | 0.000184 |
| 17 | 6 | 0 | -3.664823 | -4.078634 | 0.000250 |
| 18 | 6 | 0 | -2.343078 | -4.407466 | 0.000221 |
| 19 | 6 | 0 | 3.596860 | 1.804917 | 0.000044 |
| 20 | 6 | 0 | 2.196531 | 2.118488 | -0.000018 |
| 21 | 6 | 0 | 1.215843 | 1.089813 | 0.000132 |
| 22 | 6 | 0 | -0.163757 | 1.416084 | -0.000059 |
| 23 | 6 | 0 | -1.149169 | 0.384815 | 0.000138 |
| 24 | 6 | 0 | -2.524367 | 0.719529 | -0.000020 |
| 25 | 6 | 0 | -3.526821 | -0.295704 | 0.000052 |
| 26 | 6 | 0 | -4.930462 | 0.030941 | -0.000012 |
| 27 | 6 | 0 | -5.858167 | -1.025685 | 0.000121 |
| 28 | 6 | 0 | -5.453903 | -2.356028 | 0.000221 |
| 29 | 6 | 0 | 4.517620 | 2.846364 | 0.000082 |
| 30 | 6 | 0 | 4.103155 | 4.193562 | 0.000101 |
| 31 | 6 | 0 | 2.764056 | 4.522494 | 0.000080 |
| 32 | 6 | 0 | 1.783341 | 3.495666 | 0.000057 |
| 33 | 6 | 0 | 0.410330 | 3.805201 | 0.000061 |
| 34 | 6 | 0 | -0.557074 | 2.799782 | 0.000043 |
| 35 | 6 | 0 | -1.951955 | 3.109672 | 0.000047 |
| 36 | 6 | 0 | -2.924815 | 2.106551 | 0.000013 |
| 37 | 6 | 0 | -4.316373 | 2.403755 | -0.000048 |
| 38 | 6 | 0 | -5.296969 | 1.444965 | -0.000100 |
| 39 | 8 | 0 | 5.106877 | 5.125530 | 0.000141 |
| 40 | 6 | 0 | -6.677245 | 2.020735 | -0.000269 |
| 41 | 8 | 0 | -4.670225 | -5.005775 | 0.000344 |
| 42 | 8 | 0 | -6.912252 | 3.218941 | 0.000255 |
| 43 | 8 | 0 | -7.698581 | 1.128647 | -0.001269 |
| 44 | 6 | 0 | 7.160955 | -1.755484 | -0.000156 |
| 45 | 8 | 0 | 7.564957 | -2.903047 | -0.000160 |

| | | | | | |
|---|---|---|---|---|---|
| 46 | 8 | 0 | 8.016409 | -0.697388 | -0.000198 |
| 47 | 8 | 0 | 2.864539 | -4.301961 | -0.000080 |
| 48 | 8 | 0 | -2.249663 | 4.436160 | 0.000105 |
| 49 | 1 | 0 | 6.132311 | 0.753921 | -0.000121 |
| 50 | 1 | 0 | 5.077489 | -3.385557 | -0.000095 |
| 51 | 1 | 0 | 0.281257 | -4.764154 | 0.000155 |
| 52 | 1 | 0 | -2.036070 | -5.451493 | 0.000265 |
| 53 | 1 | 0 | -6.914443 | -0.808181 | 0.000137 |
| 54 | 1 | 0 | -6.199429 | -3.142612 | 0.000320 |
| 55 | 1 | 0 | 5.585151 | 2.664893 | 0.000116 |
| 56 | 1 | 0 | 2.443507 | 5.561782 | 0.000100 |
| 57 | 1 | 0 | 0.097481 | 4.842809 | 0.000106 |
| 58 | 1 | 0 | -4.670217 | 3.430526 | -0.000082 |
| 59 | 1 | 0 | 4.731519 | 6.016018 | 0.000174 |
| 60 | 1 | 0 | -4.295767 | -5.896607 | 0.000395 |
| 61 | 1 | 0 | -8.514214 | 1.658415 | -0.001330 |
| 62 | 1 | 0 | 8.914302 | -1.068237 | -0.000228 |
| 63 | 1 | 0 | 2.131074 | -4.928771 | -0.000248 |
| 64 | 1 | 0 | -3.204764 | 4.579969 | 0.000215 |

**Table S3. Standard orientation of TCBQ.**

| Center | Atomic | Atomic | Coordinates (Angstroms) | | |
|---|---|---|---|---|---|
| Number | Number | Type | X | Y | Z |
| 1 | 6 | 0 | -0.000011 | 1.456176 | -0.000107 |
| 2 | 6 | 0 | -1.278309 | 0.674806 | 0.000004 |
| 3 | 6 | 0 | -1.278308 | -0.674814 | 0.000118 |
| 4 | 6 | 0 | 0.000010 | -1.456177 | 0.000383 |
| 5 | 6 | 0 | 1.278309 | -0.674807 | 0.000093 |
| 6 | 6 | 0 | 1.278307 | 0.674812 | -0.000020 |
| 7 | 8 | 0 | -0.000010 | 2.670977 | 0.000132 |
| 8 | 8 | 0 | 0.000008 | -2.670977 | -0.000122 |
| 9 | 17 | 0 | -2.719917 | 1.617127 | -0.000119 |
| 10 | 17 | 0 | -2.719894 | -1.617145 | 0.000089 |

| | | | | | |
|---|---|---|---|---|---|
| 11 | 17 | 0 | 2.719918 | -1.617126 | 0.000033 |
| 12 | 17 | 0 | 2.719895 | 1.617146 | -0.000174 |
